# Supplementary figures and images for: Healthcare workers’ beliefs, practices and experiences regarding the COVID-19 pandemic and resulting governance from preparedness and response plans in Faranah, Guinea
Source: PLOS Glob Public Health. 2026 Mar 27;6(3):e0004848. doi: 10.1371/journal.pgph.0004848 (PMC13029724; doi:10.1371/journal.pgph.0004848)

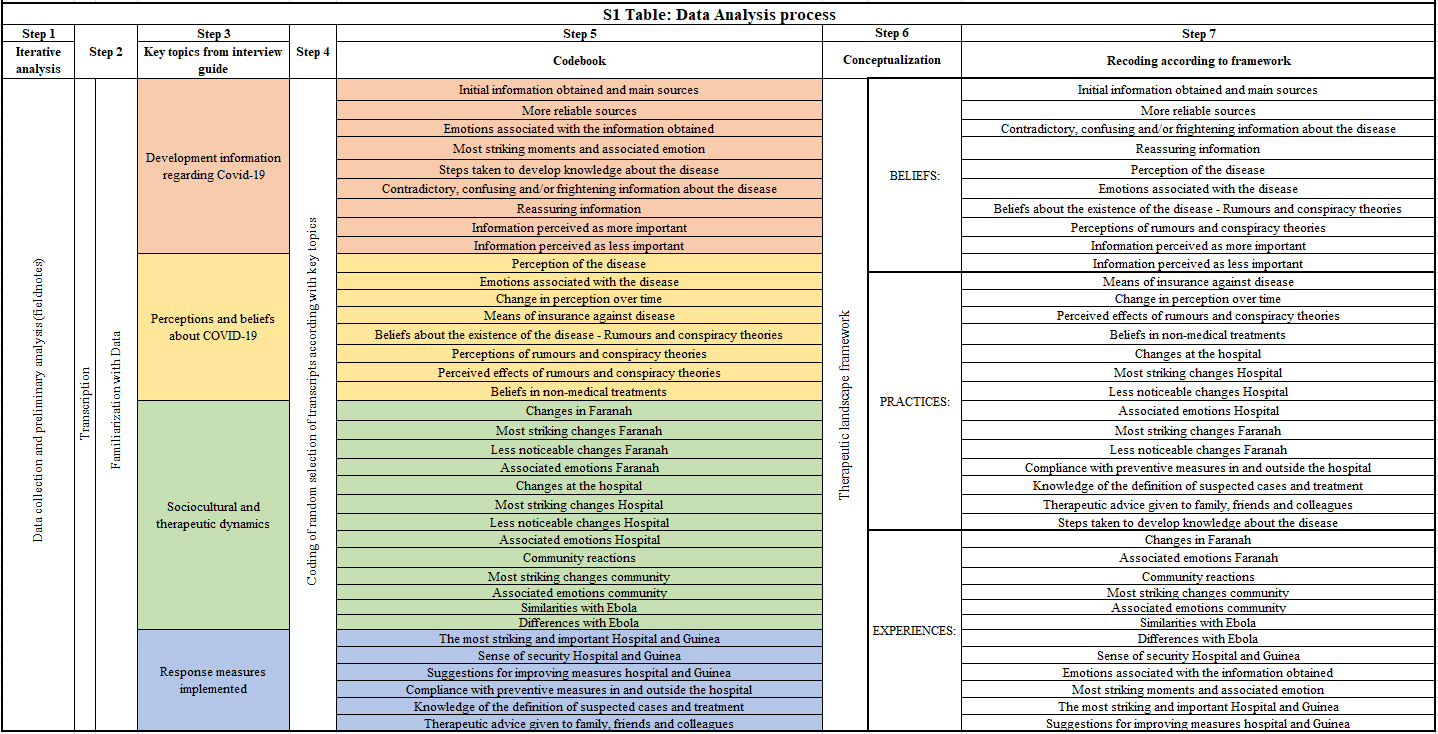

Supplement: S1 Table — (DOCX) [file pgph.0004848.s001.docx]
