# Supplementary material for: Healthcare workers’ beliefs, practices and experiences regarding the COVID-19 pandemic and resulting governance from preparedness and response plans in Faranah, Guinea
Source: PLOS Glob Public Health. 2026 Mar 27;6(3):e0004848. doi: 10.1371/journal.pgph.0004848 (PMC13029724; doi:10.1371/journal.pgph.0004848)
Supplement: Attachment — Submitted filename: Response to reviewers.docx [file pgph.0004848.s003.docx]

**Reviewer response letter:**

Dear staff editor and reviewers,

On behalf of all the co-authors from the manuscript number PGPH-D-25-01478 “Healthcare Workers’ Beliefs, Practices and Experiences regarding the COVID-19 Pandemic and resulting Governance from Preparedness and Response Plans in Faranah, Guinea”, I want to express our deepest appreciation for taking the time to share your experienced insights. Your comments are extremely helpful and have certainly improved the manuscript and the article’s key messages. Below are our responses to all of your comments organized by section, as well as detailed line-by-line descriptions of resulting edits to the manuscript. We hope that our revisions address all of your concerns clearly and completely. We are happy to rework the manuscript further if you feel there are still crucial elements missing.

Yours sincerely,

CarlosRocha (corresponding author)

Journal requirements:

- Attached filled-out version of questionnaire on inclusivity in global research.

Comments to the author:

- Data availability statement has been added to the manuscript (lines 650-655).

Review comments to the author:

**Reviewer #1:**

General comment:

Overall, I think this is a relevant paper. It uses a novel approach to understand the experiences of health workers during the COVID-19 pandemic. I think readers can learn how to conduct social science research during responses to disease outbreaks from the work presented in this paper.

*Thank you for finding the results from the paper relevant and for accurately grasping our intention regarding the use of social sciences methods and frameworks to study outbreak preparedness and response plans, implementation and local uptake.*

Minor comment:

Minor comment: Please change the tense in the entire paper from past participle to past tense since the pandemic was 5 years ago now. From phrases like “the COVID-19 pandemic has highlighted” to “the COVID-19 pandemic highlighted”.

*Tense has been changed to past tense across the whole manuscript.*

Methods:

1. Please state the specific study design for this study. See here: Tenny S, Brannan JM, Brannan GD. Qualitative study. From your methods I see that the specific design was grounded theory.

*Specific study design has been added in line 113-114.*

2. Please provide a definition for a health workers. Were janitors and cleaners for example eligible to participate? This cadre work in hospitals and are as likely to contract nosocomial infections just as much as other health workers.

*Definition for healthcare worker has been added in lines 121-122. The non-inclusion of janitors and cleaners as study participants was already included in the strengths and limitations section in lines 611-615.*

3. Please add the limitations of using convenience sampling as opposed to purposive sampling.

*Limitations of using convenience sampling as opposed to purposive sampling have been added in the participant selection subsection, lines 126-132.*

4. Please add a couple of sentences on how the authors ensured trustworthiness. See more here: Elo S, Kääriäinen M, Kanste O, Pölkki T, Utriainen K, Kyngäs H. Qualitative content analysis: A focus on trustworthiness. SAGE open. 2014 Feb 5;4(1):2158244014522633.

*A new subsection regarding techniques to enhance trustworthiness has been added, line 177. Information from the “data collection” subsection was moved to the new subsection and more detail was provided, lines 178-182.*

5. The data analysis section appears detailed and well described.

*Thank you!*

6. Please provide more detail about the ethical issues in the study. Was informed consent obtained? Was it verbal or written? How about administrative approval? How about approval from the hospital management?

*More detail on the ethical issues in the study has been added, please refer to lines 136-141.*

7. I think the authors should think a little bit deeper about the participant selection. They state that the exclusion criteria were anyone below 18 years or did not provide consent. 1) I don’t think you are going to find a health worker who is below 18 years. 2) Lack of consent cannot be an exclusion criterion because they would not have agreed to participate in the first place. I think there is detail lacking there.

*Information on inclusion criteria has been added, please refer to lines 122-123.*

8. Please provide more information about the interview guide. How was it developed? Was it based on a theory or framework? Please cite this.

*Further information on the interview guide has been added, lines 150-152.*

9. Data collection: Please state that the interviews were held in a language comfortable to the interviewee not the interviewer.

*Information on language from the interviews has been corrected in line 145-146.*

10. I would advise the authors to use the COREQ checklist to ensure all the items are reported for a qualitative study: <https://www.equator-network.org/reporting-guidelines/srqr/>

*Thank you for the recommendation! After thorough study of the suggested checklist, a review of the items presented in the manuscript and the incorporation of the suggestions from the reviewers concerning the methods section, we believe the new version of the manuscript complies with all the items from the checklist.*

Results:

11. The presentation of results can be smarter. Please remove the quote from the text in the paragraphs so that they are clearly visible (for example lines 230 – 235). I see that the font size is also different.

*Quotes within the text have been removed to make them more visible and font size has been streamlined. Quotes with 16 words or less have been kept in the text.*

12. The authors said that they did the analysis inductively. But from the results I see that the results are presented by category – beliefs, experiences and practices. Please clarify whether the approach was indeed inductive because the results presentation shows that the themes were generated deductively across pre-defined categories of beliefs, experiences and practices.

*More information in the “data analysis” subsection has been included to clarify the data analysis approach and strategy. Please refer to lines 158-176.*

13. Please move the definition of practice to the methods. Also, I would add definitions for the other categories -beliefs and experiences.

*Definitions for the three categories — beliefs, practices and experiences — were originally found at the beginning of each of the “results” subsections. The three definitions have now been moved to the methods section at the bottom of the “data analysis” subsection, lines 173-176.*

Discussion:

14. Could you please add a reflexivity statement in the strengths and limitations. How did the personal biases of the authors influence the findings in this work?

*A reflexivity statement has been added in the “strengths and limitations” section, please see lines 615-628.*

**Reviewer #2:**

General comment:

This is a useful qualitative analysis for the African healthcare context in public health emergencies.

*Thank you!*

1. What is also reassuring for me is when, out of 100 sick people, we say that 80 to 90 have been cured, or out of 100 people, there have only been 2 deaths, that is really reassuring’ (IDI 9). They also perceived information on the development of vaccines as encouraging.

- This should be discussed as under ascertainment of mild cases have impacted the severity perception very much in the early pandemic.

*The appraisal of mild cases as indicators of the severity perception on the pandemic has been added in the discussion, please refer to lines 512-515.*

2. According to them, rumor sources include "word of mouth", the radio, print and social media"

- Can be further discussed in light of recent evidence of social media as a main targeted mass disinformation and emotional manipulation initial source, eventually promoted by opposition parties to capitalize politically on the pandemic as referred in the next sentence. It will become more important to understand these patterns in global public health emergencies.

*The role of social media during pandemics in light of recent social sciences literature on the topic has been further discussed, please see lines 549-572.*

3. The authors should discuss Infodemic and Infodemics monitoring and management as referred in literature.

*Infodemics management and monitoring have been further discussed in light of recent social science literature on the topic from Guinea and the West African region. Please refer to lines 539-589.*

4. "Participants consistently linked the rise of these rumors and conspiracy narratives with the country’s political situation as the pandemic coincided with 2020 presidential elections." -

"4. People-centered approaches and other preparedness and response measures should become operational”

- A specific subchapter for desinformation and infodemic management with further discussion could be useful and facilitate reading. Discussing the role of social media as infodemic source in Guinea and Africa can be useful. Can it be the origin of rumors even if many people do not have smartphones or low literacy? What is the alphabetization and smartphone use in Guinea?

*A subsection “disinformation and infodemic management” has been added to the section of the discussion where the operationalization of preparedness and response measure are addressed, see please line 533.*

*Within this subsection, the following new discussion points were included:*

*- Lines 539-543: The importance of basing infodemic management on local dynamics and under a comprehensive approach.*

*- Lines 544-549: The role of traditional media and other social interactions influencing health-related decision making.*

*- Lines 549-571: the link of social media, rumors and misinformation and the role of conspiracy theories during epidemics in West Africa.*

*- Lines 572-589: social tensions and cleavages in Guinea and the role of conspiracy theories of political nature in light of the country’s epidemic history.*

3. We formulated the themes discussed in this paper in the context of a broader study on pandemic preparedness response via a HCWs’ Knowledge, Attitudes and Practices research. - is it published? I wouldn´t mind reading a mixed methods article but understand if it could become too long.

*Information on the article with the HCWs’ Knowledge, Attitudes and Perceptions data has been added in the “strengths and limitations” section as well the rationale behind publishing the two sources of data separately. Please refer to lines 605-611.*

4. Going through the article it seems that the infodemic detection, anticipation and management is something that should be referred in the abstract and conclusions chapter.

*Further information has been added in the abstract (line 45-46) and conclusion (line 636) to highpoint the contribution of the article in infodemic detection and management.*
