# Supplementary material for: Healthcare workers’ beliefs, practices and experiences regarding the COVID-19 pandemic and resulting governance from preparedness and response plans in Faranah, Guinea
Source: PLOS Glob Public Health. 2026 Mar 27;6(3):e0004848. doi: 10.1371/journal.pgph.0004848 (PMC13029724; doi:10.1371/journal.pgph.0004848)
Supplement: Attachment — Submitted filename: Response to reviewers 19.12.2025.docx [file pgph.0004848.s004.docx]

**Reviewer response letter:**

Dear staff editor and reviewers,

On behalf of all the co-authors from the manuscript number PGPH-D-25-01478R1 “Healthcare Workers’ Beliefs, Practices and Experiences regarding the COVID-19 Pandemic and resulting Governance from Preparedness and Response Plans in Faranah, Guinea”, I want to express our deepest appreciation for taking the time to share your experienced insights. Your comments are extremely helpful and have certainly further improved the manuscript and the article’s key messages. Below are our responses to all of your comments organized by section, as well as detailed line-by-line descriptions of resulting edits to the manuscript. We hope that our revisions address all of your concerns clearly and completely. We are happy to rework the manuscript further if you feel there are still crucial elements missing.

Yours sincerely,

CarlosRocha (corresponding author)

Review comments to the author:

**Reviewer #3:**

General comment:

The manuscript is well-written, methodologically sound, and provides valuable insights into healthcare workers’ experiences during the COVID-19 pandemic in Guinea. The revisions have addressed most reviewer concerns, making the paper suitable for publication.

*Thank for your comment and your appreciation of our work. We hope this paper will see the light soon.*

**Reviewer #4:**

General comment:

Firstly, well done on a wonderful project.

*Thank you very much for seeing value in our work! We hope to have addressed all of your comments thoroughly.*

Issues to review regarding the methods:

1. How power dynamics (interviewer known to participants) might bias response?

*Previous research in-country and at the hospital has demonstrated that a certain level of familiarity between the interviewer and the respondents is beneficial in decreasing social desirability and reducing in the respondents’ fear of negative repercussions. Thanks to the familiarity established through the interviewer's knowledge of the hospital environment, and the fact that he is a medical professional himself (as is the case in our research), a trusting rapport was established, which benefited data collection and reduced bias. Please refer to lines 150-153, where we further explain the benefits of this familiarity.*

*However, we acknowledge that our research is not entirely free from potential sources of bias. For this reason, in the limitations section (lines 646-659), we fully disclose the main possible sources of bias and we explain the strategies implemented to minimize them.*

2. Whether recruitment reached saturation across professional role, gender or ward?

*By closing following up the data collection process, we ensured that our study participants were distributed evenly across the age, profession, gender and service categories. This is reflected in our participants selection approach (lines 131 – 143) and confirmed with the percentages presented in Table 1 (line 214), which show no significant outliers in these categories among our study participants. We adopted a grounded theory approach to data saturation, collecting our data iteratively until content saturation was reached, i.e. until no new information emerged within the main topics from the topic guide. Please refer to the sections “Participant Selection” (line 131), “Data Analysis” (Line 165) and “Techniques to enhance trustworthiness” (Line 186) where we provide further information on this matter.*

*In the “Limitations” section, specifically in lines 641-645, we explain that categories such as role, gender and ward were not the main focus of our research, as our aim was to promote reflection on the healthcare workers’ experiences, beliefs and practices in relation to the response to the Covid-19 pandemic.*

3. No reflection on why only 21 of 74 HCWs participated, and whether non-participants may differ systematically?

*In connection with the previous answer regarding grounded theory and data saturation, our aim was to achieve thematic saturation among the hospital’s healthcare workers, while ensuring a fair representation in terms of gender, age, profession and service. As the data was collected and analyzed, we stopped collecting data when we determined that no new information was emerging from the topics in our topic guide. Having reached thematic data saturation, we are confident that our sample size fairly represents the views of the healthcare workers at the hospital at the time of data collection.*

4. It would be nice to show how codes were actually mapped to the framework so please include a schematic or table linking themes to the framework.

*Thank you for the suggestion. We have added an annex (S1 table) in which we outline our data analysis process step-by-step. Please refer to lines 184-185 of the manuscript.*

5. Beware of risk of over-generalization. Several statements imply causal relationships that qualitative interviews cannot support due to the nature of the data being collected.

*Thank you very much for your comment. In the new version we have made clearer that we draw our conclusions from the healthcare workers we interviewed and engaged with. See for example the sentence in lines 476-478; 483-485; 504-505;536-538.*

6. Several long sentences could be shortened without losing meaning.

*We have divided sentences with more than one subject and predicate into two parts. See, for example, lines 106–109 of the introduction, lines 121–124 of the methods, lines 357–360 of the results, and lines 476–480 of the discussion. Due to the use of specific terminology in our field of study and the enumeration of principles and concepts, the text contains some sentences that exceed two lines in length.*

7. These is inconsistent tense switching between past and present.

*Thank you very much for this comment. In the new version, we have streamlined the use of the past tense. Please refer to the version with track changes, in which all tenses switches are highlighted. In some sections, particularly in the discussion, we have kept the present tense to present general facts and express conclusions from others and our ourselves to round up our argument (see for example lines 476-478; 487-489; 541-544).*

*We have followed these guidelines:*

<https://www.nature.com/scitable/topicpage/effective-writing-13815989/>

8. Minor formatting inconsistencies exist.

*We have fixed the punctuation in the table, and the typographical inconsistencies around the references.*
